# Fiber Orientation Quantification for Large Area Additively Manufactured Parts Using SEM Imaging

**DOI:** 10.3390/polym15132871

**Published:** 2023-06-29

**Authors:** Rifat Ara Nargis, David Abram Jack

**Affiliations:** Department of Mechanical Engineering, Baylor University, Waco, TX 76798, USA; rifat_nargis1@baylor.edu

**Keywords:** large area additive manufacturing, carbon fiber-reinforced polymer matrix composite, fiber orientation characterization, method of ellipses, scanning electron microscopy

## Abstract

Polymer-based additively manufactured parts are increasing in popularity for industrial applications due to their ease of manufacturing and design form freedom, but their structural and thermal performances are often limited to those of the base polymer system. These limitations can be mitigated by the addition of carbon fiber reinforcements to the polymer matrix, which enhances both the structural performance and the dimensional stability during cooling. The local fiber orientation within the processed beads directly impacts the mechanical and thermal performances, and correlating the orientation to processing parameter variations would lead to better part quality. This study presents a novel approach for analyzing the spatially varying fiber orientation through the use of scanning electron microscopy (SEM). This paper presents the sample preparation procedure including SEM image acquisition and analysis methods to quantify the internal fiber orientation of additively manufactured carbon fiber-reinforced composites. Large area additively manufactured beads with 13% by weight large aspect ratio carbon fiber-reinforced acrylonitrile butadiene styrene (ABS) pellets are the feedstock used in this study. Fiber orientation is quantified using the method of ellipses (MoE), and the spatial change in fiber orientation across the deposited bead cross-section is studied as a function of process parameters including extrusion speed, raster height, and extrusion temperature zones. The results in the present paper show the results from the novel use of SEM to obtain the local fiber orientation, and results show the variation in alignment within the individual processed bead as well as an overall aligned orientation state along the direction of deposition.

## 1. Introduction

Polymer composites are seeing increased use in the manufacturing industry because of their enhanced mechanical and thermal properties, low densities, formability, and ability to mass-produce components. Of interest in the present scope is the use of short fiber-reinforced thermoplastic polymer composites to enhance structures fabricated through an additive manufacturing (AM) process. Additive manufacturing (AM), which is often termed freeform fabrication or 3D printing, is a process in which parts are built by depositing materials such as plastic, composite, metal, ceramic, and concrete, layer by layer, according to digital 3D design data. There are many variations of different additive manufacturing processes, among which fused deposition modeling (FDM) is one of the more commonly employed AM methods. Fused deposition modeling, also known as fused filament fabrication (FFF), is an additive manufacturing process that often uses thermoplastic materials such as acrylonitrile butadiene styrene (ABS), nylon, and polylactic acid (PLA), often in the form of continuous filament, fed from a heated nozzle or printer extruder head and methodically deposited to form layers. The large area additive manufacturing (LAAM) system used for this study would be considered within the family of fused deposition modeling. This system was first studied at Oak Ridge National Laboratory, where Duty et al. [1] demonstrated the ability to take raw fiber-filled pellets and fabricate large-volume AM structures. A review by Vicente et al. [2] studied recent applications for LAAM systems, showed their use in the transportation industry, and concluded that the LAAM process may be the most suitable approach for the production of large-scale components. Viciente et al. highlight that LAAM is used in the aerospace industry for reducing complex assemblies, large-scale mock-ups, and speeding the design process for new systems. In the automotive industry, Viciente et al. show use cases for LAAM for electric vehicles, prototyping, and tooling for large components. The system utilized in the present scope of work utilizes a pellet-fed system with a high-flow-rate extruder capable of ~25 lbs/h deposition. These pellet-fed systems allow for higher deposition rates while simultaneously reducing the cost of the raw materials. Recently, Pricci et al. [3] presented a sophisticated model to correlate the screw speed for a pellet-fed system similar to that of Duty et al. to that of the desired mass flow rate. Similarly, Barera et al. [4] studied the impact of the temperature and extrusion pressure on the final part performance for carbon fiber-filled polyamide 6. Yeole [5] used the LAAM process to fabricate large-scale molds and showed the anisotropy of the individual extrudate as well as the sensitivity of the final part performance to the choice of printing path. In the present study, we select ranges of screw speeds and adjust the translation speed of the extruder to obtain the desired cross-sectional area. A large area additive manufacturing (LAAM) printer is used to melt the thermoplastic pellet, and the molten material is deposited through an extruder. The material is then extruded to a heated build plate or platform where the resulting anisotropic material cools and hardens.

The polymer used in the present study is acrylonitrile butadiene styrene as it is broadly used in additively manufactured part production. ABS is a terpolymer that consists of butadiene rubber dispersed in a poly styrene-co-acrylonitrile matrix [6]. ABS has an improved toughness relative to many mass-produced polymer blends that require processing temperatures below 250 °C. The structural and thermal performance of ABS is limited, but the addition of fiber reinforcements, specifically carbon fibers, allows for enhanced structural performance while simultaneously reducing dimensional variations during thermal changes. Specifically, Love et al. [7] showed that the printed neat ABS had a CTE of 87 μm/°C, whereas their 13% carbon fiber-filled system had a CTE of 10 μm/°C along the deposition direction but 106 μm/°C transverse to the print direction. Lobov et al. [8] demonstrated that the incorporation of carbon fiber in their ABS filament improved the tensile strength by 30% and the fracture toughness by 20% in their 3D printed system. Wang et al. [9] studied 3D printed ABS systems with both carbon and Kevlar fiber reinforcements. They observed an improvement in both the strength and stiffness upon the incorporation of carbon fibers, whereas the Kevlar fibers had little to no effect on the stiffness and strength. Actually, the Kevlar fiber reduced the performance relative to the neat ABS system. Carbon fiber-filled thermoplastics allow for design tailoring ability at a relatively low cost, as demonstrated in [10]. Velez-Garcia et al. [11] demonstrated for an injection molded composite that the quantification of the fiber orientation and any induced porosity due to the addition of the fibers is essential to understanding the mechanical, structural, and thermal properties of the final manufactured part.

One of the most prevalent methods to measure the fiber orientation in a polymer matrix is the method of ellipses (MoE) (see, e.g., [12]). Using MoE, Velez-Garcia [13] showed the characteristic values of complete and incomplete elliptical footprints, and the resulting fiber orientations were quantified from micrographs. Hofmann et al. [14] extended the method to look at fiber flexure within the section region for fibers with an aspect ratio significantly larger than those in the present study. The method of ellipses for rigid fibers has its own limitations, specifically, the ambiguity problem where a pair of orientation states can be represented by a given elliptical footprint (see, e.g., [15]). Typically, the standard method of ellipses for circular fibers produces an ambiguity of the fiber orientation angle relative to the surface normal, causing an undetermined state for the off-diagonal components of the orientation tensor. However, two ambiguities can exist for elliptical cross-section fibers because of the additional degree of freedom of the roll about the axis of the fiber [16], which is not considered with the assumed axisymmetric fibers used in the present research.

As discussed by Goldstein [17], scanning electron microscopy (SEM) generates images of a sample by scanning the surface with electron beams. There are two types of electrons that can be captured with the system used in the present research: high-energy backscattered electrons and low-energy secondary electrons. From the reflected signal, SEM provides data on the surface topology and composition of the sample (see, e.g., [17]) and can generate three-dimensional-like images of the surface with the help of a large depth of field and shadow relief effects of the secondary and backscattered electron contrast. The JEOL SEM 6610-LV (USA headquarters in Peabody, Massachusetts) used in this study can achieve up to a 300,000× magnification in a high vacuum mode, but in the present study, it is the use of the low vacuum mode that allows for the investigation of features just below the surface of the polished sample without the need to sputter coat the surface with an electrically conductive material. The present work utilizes the electrical mismatch between the semi-conducting carbon fibers and the electrically insulating polymer matrix to yield high-resolution images of the boundary between individual carbon fibers and an ABS matrix. Additional non-optical methods were considered by the authors, such as optical-FTIR and AFM, and may be worth future studies. For example, the mismatch in stiffness between the polymer matrix and the carbon fiber may be detectable by AFM. Similarly, the difference in the IR spectrum of the polymer matrix and the carbon fiber may be identifiable using high-resolution optical-FTIR. Due to the availability of SEM to the researchers, SEM was used in this study to produce electrical surface maps to quantify the direction of each fiber in the etched surface, and the results in the present paper provide a potential alternative method to remove the orientation ambiguity.

In fiber-reinforced polymers, the volume fraction of fiber, type of fiber, fiber size, and fiber orientation directly influence the performance of the material (see, e.g., [18]). Fiber orientation in the matrix may vary due to the motion of the fiber compared to the surrounding matrix in the material flow from the nozzle during processing. Love et al. [7] demonstrated that the addition of carbon fibers into the acrylonitrile butadiene styrene (ABS) polymer matrix showed a significant increase in the stiffness and strength of their FDM 3D printed parts. The final 3D printed part bears most of the loads in the print direction where the fibers are mostly aligned, as demonstrated by Tekinalp et al. [19]. The tensile strength and tensile modulus were shown in [19,20] with the addition of carbon fibers. Specifically, in [19], the authors found that the addition of carbon fibers enhanced the tensile strength and tensile stiffness by 115% and 700%, respectively. Russell and Jack [21] obtained a stiffness of 2.25 GPa for their neat ABS system, which was increased to 3.2 GPa for their lightly loaded, moderately aligned carbon fiber-reinforced system. Similarly, Duty et al. [1] found that through the addition of carbon fibers and enhancing the elongational effects to enhance the fiber alignment, they were able to achieve a stiffness of 8.2 GPa for their carbon fiber-filled ABS system. In addition to impacting the structural performance, the fiber orientation will influence the thermal properties such as thermal conductivity and coefficient of thermal expansion. The thermal conductivity was shown by Chen and Wang [22] to be substantially higher where the fibers have a higher degree of fiber orientation for their injection-molded composite. Hassan et al. [23] showed that through the addition of carbon fibers to their ABS system, the thermal conductivity increased by a factor of 3 to 7 times that of the neat polymer as a function of orientation. In some cases, they observed that the conductivity along the aligned fiber direction was 3 to 4 times that transverse to the alignment. Tezvergil et al. [24] showed that in the direction of fiber alignment, the coefficient of thermal expansion is lower than that of the base polymer, whereas transverse to the fiber alignment direction, the coefficient of thermal expansion is closer to that of the base polymer.

In the present study, a method is presented to quantify the local fiber orientation of an additively manufactured carbon fiber-reinforced composite through the use of scanning electron microscopy. In addition, the industrially relevant LAAM-deposited beads, with a cross-sectional area greater than 20 mm^2^, are investigated, whereas most literature focuses on beads with a cross-sectional area less than 1 mm^2^. The approach in the present study also differs from that of the literature which typically relies upon optical microscopy methods. Samples are formed using the large area additive manufacturing process and are then sectioned, polished, and imaged using SEM. The SEM approach leverages the mismatch in electrical conductivity between that of the semi-conducting carbon fiber and the electrically insulating polymer matrix. In addition to presenting a new method for quantifying the local changes in orientation within the LAAM-deposited beads, several process parameters unique to the LAAM approach, specifically screw speed, processing temperature zones, and bead cross-sectional area, are studied for their impact on the final orientation state of fibers.

## 2. Materials and Methods

The following sections discuss the selection of various process parameters studied for the LAAM system, the sample preparation procedure, and the image acquisition. In total, 27 different sets of processing parameters were investigated over a range of temperature profiles in the extruder, extrusion speed, and cross-sectional area of the deposited bead. The LAAM system was custom designed by the researchers to process fiber filled pellets with the full specifications and part component listing given in [25]. In addition, six additional specimens were fabricated to correlate the sensitivity of the developed fiber orientation quantification method to the sample size.

### 2.1. Sample Fabrication

In this research, to study the effects of different process parameters on the fiber orientation, three process parameters were selected: nozzle temperature, screw speed within the extruder by varying the RPM, and the area of the cross-section of the deposited extrudate. The extruder had three different heated zones. The three set point temperatures for zones 1, 2, and 3, were set to three different scenarios, specifically 190/195/200 °C, 200/205/210 °C, and 210/215/220 °C, with the last temperature zone listed being closest to the extrusion nozzle. Three different RPM values were selected for the single screw extruder: 1500 RPM, 1875 RPM, and 2250 RPM. Lastly, the three cross-sectional areas studied were 22 mm^2^, 27 mm^2^, and 32 mm^2^, where the cross-sectional area was of the deposited bead. The extruder height relative to the print bed was kept fixed for all specimens in the present study at 3 mm from the height of the previously deposited layer. The cross-sectional area of a printed bead for a certain screw speed and a certain temperature was controlled by adjusting the gantry speed. This resulted in a total of $3^{3}=27$ samples in this study to analyze for fiber orientation. All samples in the present study were composed of 3 layers of deposited beads, with the interior bead being used for all the orientation analyses. This interior bead would be indicative of the majority of the deposited beads in a layered structure, whereas the top and bottom beads would potentially have a different orientation state due to edge effects.

To control the cross-sectional area of the final print part, the G-code controlling the gantry speed was modified. The mass flow rate ${\dot{m}}_{ABS}$ was measured using a standard laboratory scale and stopwatch for each of the 9 variations of extruder zone temperatures and extruder screw speed. The volumetric flow rate, ${\dot{V}}_{ABS}$, is found from the mass flow rate ${\dot{m}}_{ABS}$ divided by the density $\rho_{ABS}$ as follows:

(1) $${\dot{V}}_{ABS}=\frac{{\dot{m}}_{ABS}}{\rho_{ABS}}=\frac{m_{ABS}}{t\;\rho_{ABS}}$$

where ${\dot{m}}_{ABS}$ is the measured mass of extruded polymer over the duration of time, *t*. This can be converted to the speed of the gantry by dividing it by the desired cross-sectional area $A_{ABS}$ as follows:

(2) $$Gantry\;Speed=\frac{{\dot{V}}_{ABS},\;}{A_{ABS}}$$

The actual cross-sectional areas of the layers of the printed sample were validated against the estimate from Equation (2) with the help of optical microscopy of sectioned samples. The 27 samples are categorized according to the process parameters and shown in Table 1.

### 2.2. Surface Preparation for Imaging

Proper surface preparation of the sectioned samples is critical to obtain the desired characteristic material and topological data for both optical and scanning electron microscopy imaging, specifically obtaining a high contrast between the fiber edge and the surrounding polymer matrix. Sample preparation steps include 3D printing the chopped carbon fiber-filled ABS on the available LAAM system, sectioning samples using a low-speed saw, placing samples into a thermoset mold, polishing the molds to expose the sample surface, cleaning the sample surface during polishing, and etching the polished samples to expose the fiber edges.

For polishing the samples, a proper recipe is very important for obtaining the best micrographs while using microscopes. Velez-Garcia et al. [11] presented a methodology for polishing composites with glass fibers that was not effective for the carbon fiber-reinforced ABS samples used in the present study. A parametric study was performed in the present research, and a suitable polishing technique for the carbon fiber-reinforced composites was identified. The carbon fiber samples were polished with a Buehler EcoMat 3000 (headquarters, Lake Bluff, Illinois, USA) variable-speed grinder automatic polisher, with 120-, 320-, 400-, 600-, and 1200-grade silicon carbide electro-coated abrasive sandpaper. Samples were polished in a wet environment with 120-grade sandpaper for 2 min, 320-grade for 4 min, 400-grade for 6 min, 600-grade for 6 min, and 1200-grade for 12 min. Wet conditions while polishing are essential to avoid excessive fiber breakage as carbon fibers are very lightweight and the grinding from the polisher can easily break the fibers and embed them into the surrounding polymer matrix, as was observed in the present study along with the earlier work of Velez-Garcia et al. [11]. The continuous flow of the fluid, in this case, water, helps to eliminate the debris.

Next, the sample was polished with a micro-cloth with an alumina polishing compound in three stages. In each stage, a successively smaller grit of the polishing compound was used: 5-micron, 1-micron, and then 0.5-micron. Each step was performed for 30 min for each of the three polishing steps, and polishing compounds were added every 10 min. The sequence of polishing was repeated once to enhance the surface’s smoothness. In all polishing steps, a wet environment was maintained continuously to avoid excessive breakage of fibers on the surface and to enhance the exposed fiber footprint border.

High-frequency and high-intensity sound waves are used for removing foreign contaminants from the surface of a sample, as performed in [26]. During polishing, the sample was cleaned for 30 s with a Branson 1510 ultrasound sonicator (St. Louis, MO, USA), every 10 min during sanding, to remove any debris that could accumulate and scratch the surface. During sonication, an ultrasound polishing compound was mixed with water in a ratio of 6:1 to serve as a cleaning agent and rinsed in water after sonication.

**Figure 1:**
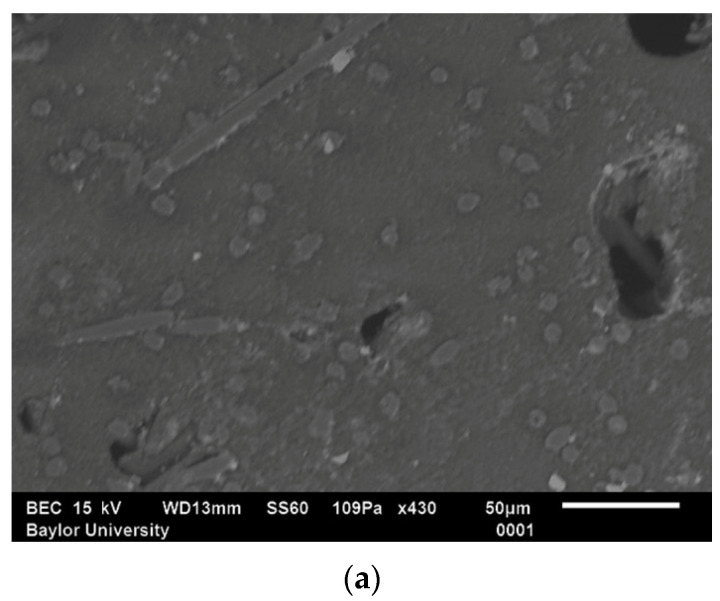

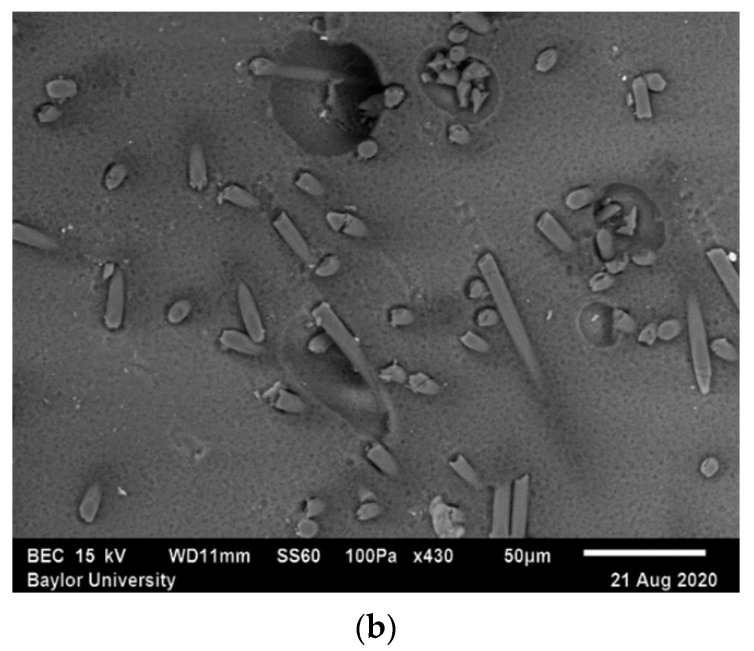
SEM images (**a**) before plasma etching and (**b**) after plasma etching a sample.

Typical SEM produces images in grayscale and often produces images of a degraded low contrast [27]. There are many image processing techniques for enhancing degraded images (see, e.g., [16,25]). In the present work, plasma etching, similar to that described in [11], was used to remove a small layer of material from the polished surface to enhance the contrast without the use of image processing. After etching, the fibers slightly stick out of the matrix, which provides a clearer distinction from the matrix. The sample was etched with a Plasma-Etch PS-50 (Carson City, NV, USA) for 40–50 min in an environment of oxygen mixed with carbon tetrafluoride (CF_4_). The ratio of oxygen and CF_4_ gas was 3:1. The before and after images taken from different regions of the same sample are provided in Figure 1, and the enhanced contrast from the small removal of the polymer matrix from the fiber perimeter can be observed. Notice that in Figure 1a there is little contrast between the carbon fibers and the surrounding polymer matrix. However, after etching, the fibers in Figure 1b are clearly evident, and their surface area is easier to quantify. For all SEM images, the system settings, including the working distance, spot size, electron voltage, and magnification, are shown alongside the scale bar. All samples in the present study were etched after the completion of the final polishing step.

### 2.3. Image Acquisition Process from Scanning Electron Microscope

A JEOL JSM-6610LV Scan Electron Microscope was used to capture high-resolution images with an increased depth of field of the polished sample using the available backscatter detection mode.

**Figure 2:**
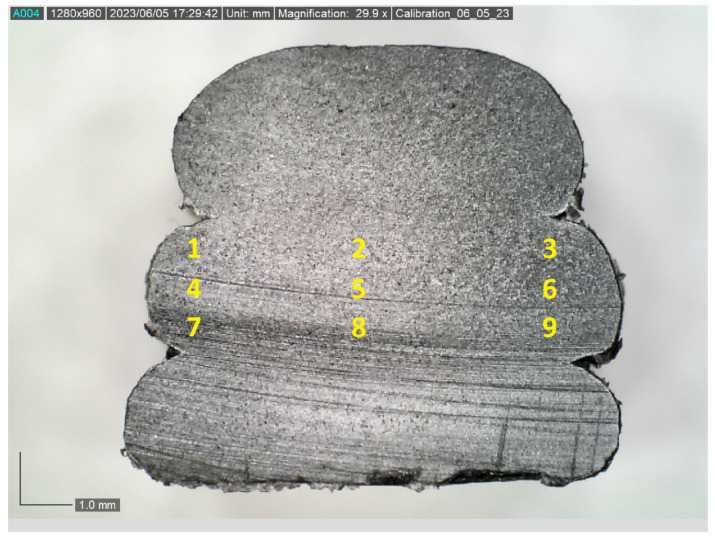
Optical image of the surface of the polished sample taken at ~30× magnification.

In Figure 2, an optical image of the polished sample is shown at 20× magnification. Observe in the figure that there are three deposited layers, termed beads. The sample is from a custom large area additively manufactured system as described in Russell [25]. The typical fiber length produced in this system is on the order of 200~300 μm with a nominal aspect ratio ranging from 20 to 40, much larger than that of standard fused filament systems with fiber aspect ratios ranging from 5 to 10. The larger-aspect-ratio fibers provide a significant improvement in the final processed part performance. In the present study, we will focus on the middle bead as that would be representative of the core region of the fabricated component. This middle bead is further subdivided into 9 sub-regions as depicted in Figure 2, and the results for the orientation state within each region will be quantified in the following results. Later results will show that the center section of the bead, Region 5, has the lowest alignment state, whereas the corner regions of the deposited bead, Regions 1, 3, 7, and 9, tend to have the highest alignment state.

**Figure 3:**
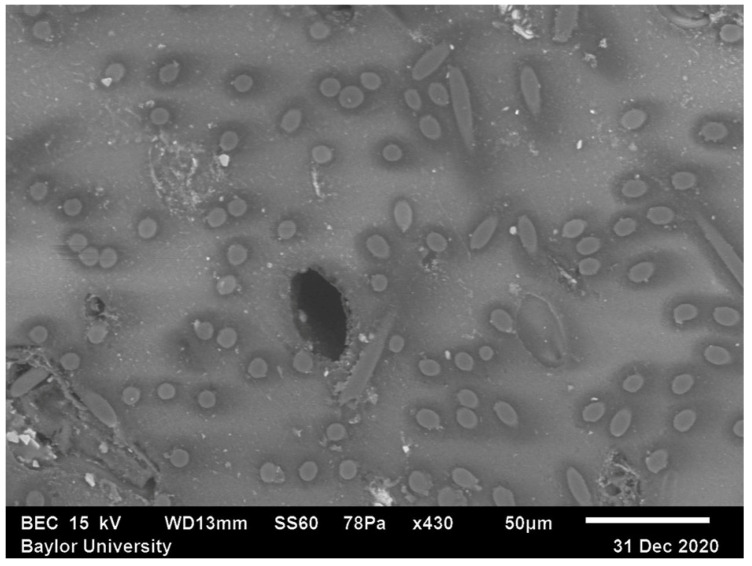
SEM Image of Region 1.

In Figure 3, the image acquired from the scanning electron microscope of Region 1 from Figure 2 is shown. In Region 1, the fibers are mostly oriented towards the out-of-plane direction, defined as $x_{3}$ (into and out of the page). The fibers are the regions of the circular or near-circular ellipses. Notice in the figure several small areas where the signal appears to be saturated, indicated by white. These regions are caused by poor surface conductivity and were identified to be the loose alumina polishing compound entrapped within voids that were exposed during polishing. The dark region in the center of the image is a large void within the extrudate. The smaller voids that are shown by the entrapped alumina and the macro voids will both contribute to a reduction in the overall structural performance, whereas the larger voids tend to randomize the final alignment state.

**Figure 4:**
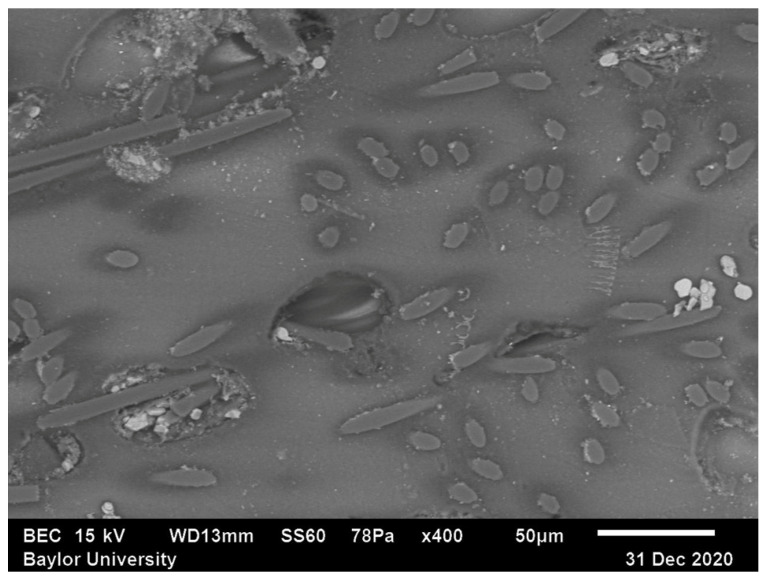
SEM Image of Region 5.

Shown in Figure 4 is the SEM image of the center-middle region of the sample extruded bead depicted in Figure 2 and identified as Region 5. In contrast to Region 1, in Region 5, the fibers are not uniformly dispersed but have a banded nature to the dispersion. In addition, the ellipsoids observed have a larger major-to-minor axis ratio indicating less alignment in the flow direction. This latter observation is made based on viewing the variations in the cross-sectioned ellipses. It is also clear that there are several significant voids measurably larger than the fibers themselves as well as multiple voids on the order of the fiber diameters present throughout the observed region. The SEM images of all 9 regions for each of 27 samples defined in Table 1 were collected as part of this study and were used in the present work for the fiber orientation quantification presented in a later section.

## 3. Fiber Orientation Identification

### 3.1. Fiber Orientation Measurement

**Figure 5:**
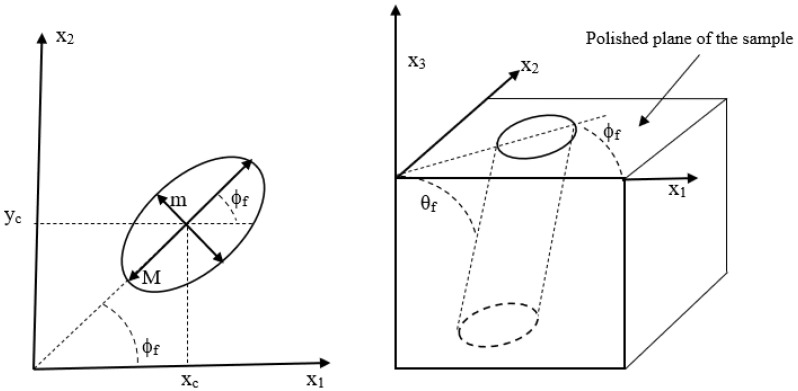
Definition of geometrical parameters measured in the method of ellipses.

Fiber orientation measurements in the composite material are very important for a reliable assessment of the physical properties. Reflective optical microscopy and scanning electron microscopy are two common methods for fiber orientation measurement (see, e.g., [28]). For fibers with a circular cross-section, the most common method for the quantification of fiber orientation is the method of ellipses (MoE) (see, e.g., [4,19,20]). For the circular fibers, at the cross-section which appears on the intercepting plane, an elliptical image can be observed, as shown in Figure 5. The directional angles $\left(\theta_{f},\;\phi_{f}\right)$ of the ellipse are shown in Figure 5 along with the center of the ellipse $\left(x_{c},\;y_{c}\right)$ and the major M and minor m axes of the ellipse.

**Figure 6:**
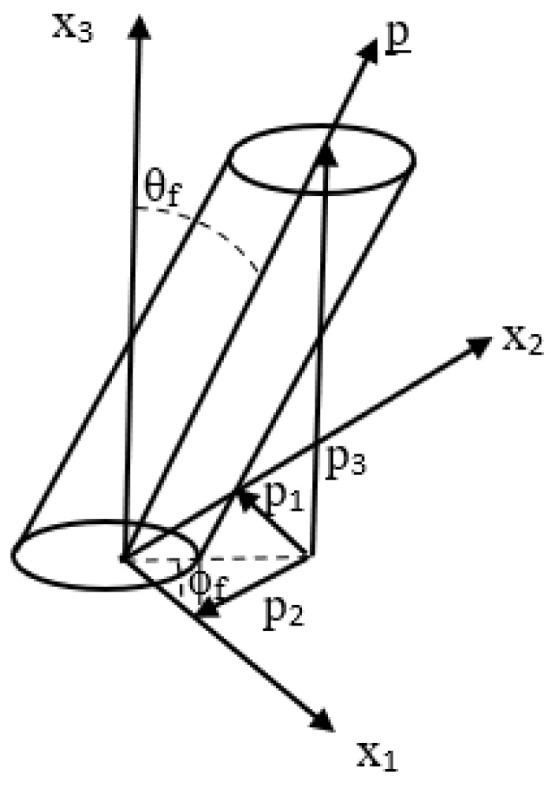
Rigid fiber particle orientation in spherical coordinates along with the polished plane from sectioning indicated by the top surface.

The out-of-plane angle, $\theta_{f},$ can be obtained from the geometry as

(3) $$\theta_{f}=\cos^{-1}\left(\frac{m}{M}\right)$$

where M is the major axis, m is the minor axis, and $\theta_{f}$ and is the out-of-plane angle (see, e.g., [15]). Short or chopped fibers in a composite matrix flow behave as rigid cylindrical rods. Short fibers can translate and rotate in the polymer melt in any of the three coordinate directions. For the LAAM system considered, with aspect ratios ranging between 20 and 40, the fibers were not observed to have any flexure. For a single fiber with a high aspect ratio, the spatial orientation can be described using spherical coordinates with the in-plane angle $\phi_{f}$, where $\phi_{f}\in \left\{0,2\pi \right\}$, and out-of-plane angle $\theta_{f}$, where $\theta_{f}\in \left\{0,\pi \right\}$, as shown in Figure 6. The unit vector p, obtained from Figure 6, has the following components:

(4) $$p=\left\{\sin\theta_{f}\cos\phi_{f},\sin\theta_{f}\sin\phi_{f},\cos\theta_{f}\;\right\}$$

Orientation tensors were popularized by Advani and Tucker [29] to represent the orientation of a population of fibers composed of the individual orientations of each individual fiber. The local orientations of the fibers in a part are commonly represented by the orientation tensors. For example, the second-order orientation tensor $A_{ij}$ is the probability density of the inner product of direction vector p with itself. For a continuous distribution of fibers defined by the orientation probability density function $\psi \left(\phi_{f},\theta_{f}\right)$, this is defined as follows:

(5) $$A_{ij}=\int_{S}p_{i}\left(\theta_{f},\phi_{f}\right)p_{j}\left(\theta_{f},\phi_{f}\right)\psi \left(\theta_{f},\phi_{f}\right)dS$$

where S is the unit sphere. By construction, orientation tensors are symmetric, $A_{ij}=A_{ji}$, and their trace is unity, $A_{11}+A_{22}+A_{33}=1$. For a discrete selection of fiber angles randomly selected from the orientation probability density function $\psi \left(\phi_{f},\theta_{f}\right)$, such as the case when measuring the orientation from the SEM images in the present study, the orientation tensor $A_{ij}$ may be expressed as follows (see, e.g., [15]):

(6) $$A_{ij}\approx \frac{\sum_{n=1}^{N_{f}}{\left(p_{i}p_{j}\right)}_{n}L_{n}F_{n}}{\sum_{n=1}^{N_{f}}L_{n}F_{n}}$$

where $L_{n}$ is the length of the nth fiber and $N_{f}$ is the total number of fibers in the sample. The expression $F_{n}$ is a weighing function, defined in terms of $L_{n}$ and the diameter of the nth fiber $d_{n}$, which relates the orientation per unit area to the orientation per unit volume and is defined as

(7) $$F_{n}=\frac{1}{L_{n}\cos{\left(\theta_{f}\right)}_{n}+d_{n}\sin{\left(\theta_{f}\right)}_{n}}$$

In the present study, the value for the length is 250 µm and the diameter is 7 µm; both values are taken from the companion study of Russell [25].

**Figure 7:**
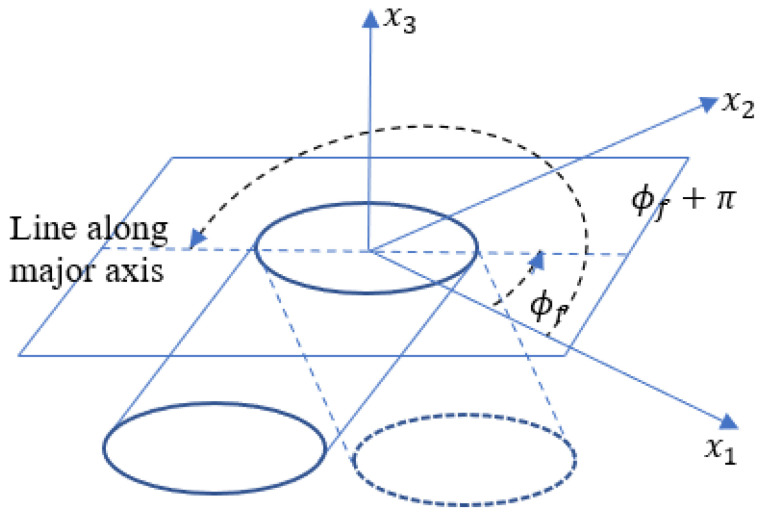
Depiction of sectioned surface demonstrating the fiber ambiguity problem.

The method of ellipses has some experimental and geometrical limitations that can lead to inaccurate measurements (see, e.g., [28,30]) which may lead to inaccurate interpretations of the fiber orientation state. When using standard optical techniques for the data collection, there will be a problem of ambiguity for the out-of-plane angle $\theta_{f}$ due to the fiber symmetry, but by construction, $\psi \left(\phi_{f},\theta_{f}\right)$ retains the same symmetries, so this is not an issue. Of experimental concern is the sensitivity in the measurement of $\theta_{f}$ which increases as the cross-section of fiber in the prepared specimen approaches that of a perfect circle, resulting in measurement errors (see, e.g., [28]). This error can be mitigated by increasing the resolution of the images used for analysis. A third source of potential error is termed the ambiguity problem and is depicted in Figure 7 and shown by two fibers, one with $\phi_{f}$ and a second with $\phi_{f}+\pi$. Notice in this case that both fibers have identical cross-sections in the $\left(x_{1},x_{2}\right)$ plane, and this ambiguity for $\phi_{f}$ prevents the tabulation of off-diagonal components of $A_{ij}$, $A_{13}$ and $A_{23}$, from being computed. As the diagonal components of $A_{ij}$ are products of the unit vector $p_{i}$ with itself, the choice of $\phi_{f}$ or $\phi_{f}+\pi$ does not change the result.

Velez-Garcia et al. [11] developed a method for solving the ambiguity issue by exposing the polished fiber tips from underneath the polished surface by plasma etching of glass fiber samples. This exposes a characteristic shadow in the optical reflection micrograph in the under-surface region and was used to determine the direction of angle alignment [11]. With SEM, a similar shadow or tail will be shown below both with and without plasma etching for semi-conducting fibers, such as carbon fibers. Effective visualization of the shadow is very important to determine the orientation of the fiber in the matrix.

Another important aspect for measuring the fiber orientation is the flexibility of carbon fibers in the ABS matrix. The flexibility is described as the tendency of the fibers to bend in the material flow (see, e.g., [31,32]). After analyzing all the SEM images in this study and in the companion study of Russell [25], no bending of the fiber in the investigated system was found.

### 3.2. Fiber Orientation Quantification

As shown in Equation (3) the orientation of the fiber can be determined in terms of the semi-major and semi-minor axis lengths. Each fiber is traced from the image created using SEM by capturing multiple points $\left(x_{1},x_{2}\right)$ on the perimeter of each fiber. These points are then used to fit, in a least-squares sense, the equation for an ellipse as

(8) $$ax_{1}{}^{2}+2bx_{1}x_{2}+cx_{2}{}^{2}+2dx_{1}+2fx_{2}+g=0$$

**Figure 8:**
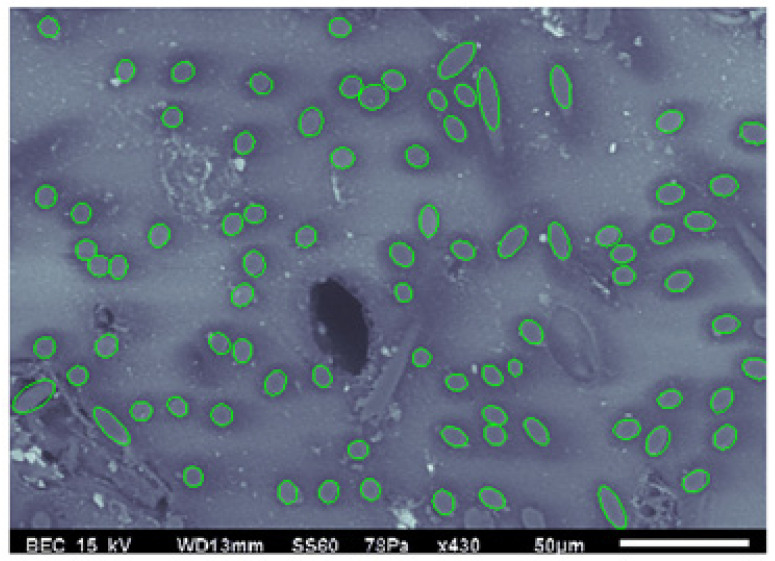
Best-fit ellipses from SEM image of Region 1.

The ellipses are drawn around the cross-section from the data as shown in Figure 8 by clicking at least 6 points to define the perimeter, and for the objects with higher ellipticity, up to 15 points were used to define the perimeter. An optimization algorithm using least-squares regression was created in MATLAB (Headquarters at Natlick, MA, USA) to estimate the parameters a, b, …, g. Once they are tabulated, the center point ($x_{1,0},\;x_{2,0})$ along with the semi-major axis M and semi-minor axis m, can be calculated as

(9) $$x_{1,0}=\frac{cd-bf}{b^{2}-ac}$$

(10) $$x_{2,0}=\frac{af-bd}{b^{2}-ac}$$

(11) $$M=\sqrt{\frac{2\left(af^{2}+cd^{2}+gb^{2}-2bdf-acg\right)}{\left(b^{2}-ac\right)\left[\sqrt{{\left(a-c\right)}^{2}+4b^{2}}-\left(a+c\right)\right]}}$$

(12) $$m=\sqrt{\frac{2\left(af^{2}+cd^{2}+gb^{2}-2bdf-acg\right)}{\left(b^{2}-ac\right)\left[-\sqrt{{\left(a-c\right)}^{2}+4b^{2}}-\left(a+c\right)\right]}}$$

Lastly, the rotation from the $x_{1}$ axis to the major axis, specifically angle $\phi_{f}$ from Figure 7, is cast in terms of the elliptical parameters as

(13) $$\phi_{f}=\{\begin{array}{cc} 0 & for\;b=0\;\mathrm{and}\;a<c\\ \frac{1}{2}\pi & for\;b=0\;\mathrm{and}\;a>c\\ \frac{1}{2}cot^{-1}\left(\frac{a-c}{2b}\right) & for\;b\neq 0\;\mathrm{and}\;a<c\\ \frac{\pi }{2}+\frac{1}{2}cot^{-1}\left(\frac{a-c}{2b}\right) & for\;b\neq 0\;\mathrm{and}\;a>c \end{array}$$

Figure 8 shows the elliptical curve fits over each individual cross-sectioned fiber from Region 1, the same data previously shown in Figure 3. The semi-major and semi-minor axis lengths along with the direction of the major axis are extracted from the best-fit ellipse to Equation (8), and this information is then used to evaluate the out-of-plane angle $\theta_{f}$ from Equation (3) for each individual fiber. With the in-plane and out-of-plane angle values, the unit vector p is evaluated from Equation (4), and then the orientation tensor $A_{ij}$ is evaluated from Equation (6).

## 4. Orientation Results and Discussion

**Figure 9:**
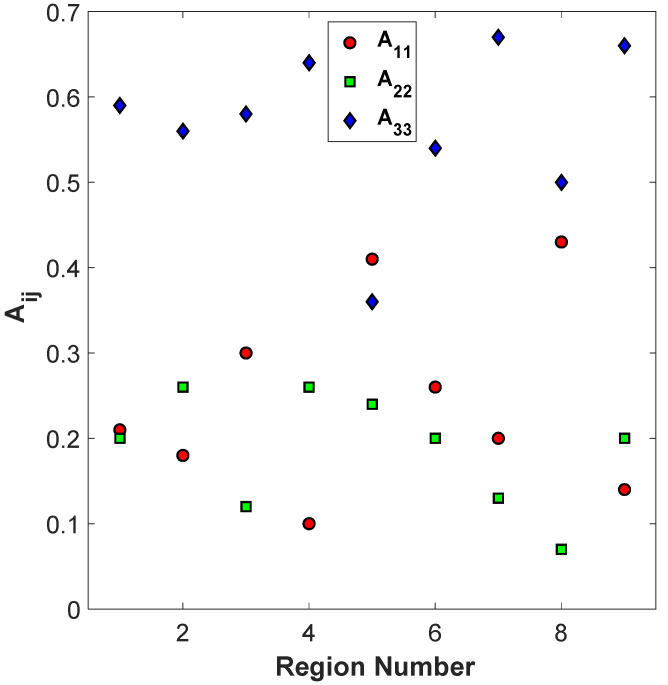
Change in $A_{11}$, $A_{22}$, and $A_{33}$ in 9 different regions of a layer of sample 15 in the deposited bead.

SEM imaging and the elliptical analysis for orientation were performed on all 27 samples from Table 2 for each of the nine regions shown in Figure 2 for a total of 243 unique datasets, with each dataset containing between 50 and 95 identifiable elliptical cross-sections. One sample, sample 15, was randomly selected for an expanded analysis. The average for the orientation parameters $A_{11}$, $A_{22}$, and $A_{33}$ along with the respective fiber count is provided in Table 2 for just sample 15. The orientation data from Table 2 are also plotted in Figure 9.

From the fiber orientation measurement given in Table 2 and shown in Figure 9, it is evident that fibers are most aligned towards the $x_{3}$ direction as $A_{33}$ is generally the highest parameter, but there are significant spatial inhomogeneities of the orientation state within the individual deposited bead. Notice that for regions along the vertical centerline, Regions 2, 5, and 8, the alignment tends to be lower (lowest $A_{33}$), as is the case for the regions along the horizontal centerline, Regions 4, 5, and 6, with the center region of the extrudate, Region 5, experiencing the lowest alignment along the print direction. Although Region 2 has a relatively high alignment in this particular sample, Region 2 has a relatively lower fiber alignment in general for the majority of the samples investigated, which will be shown later in this text.

**Figure 10:**
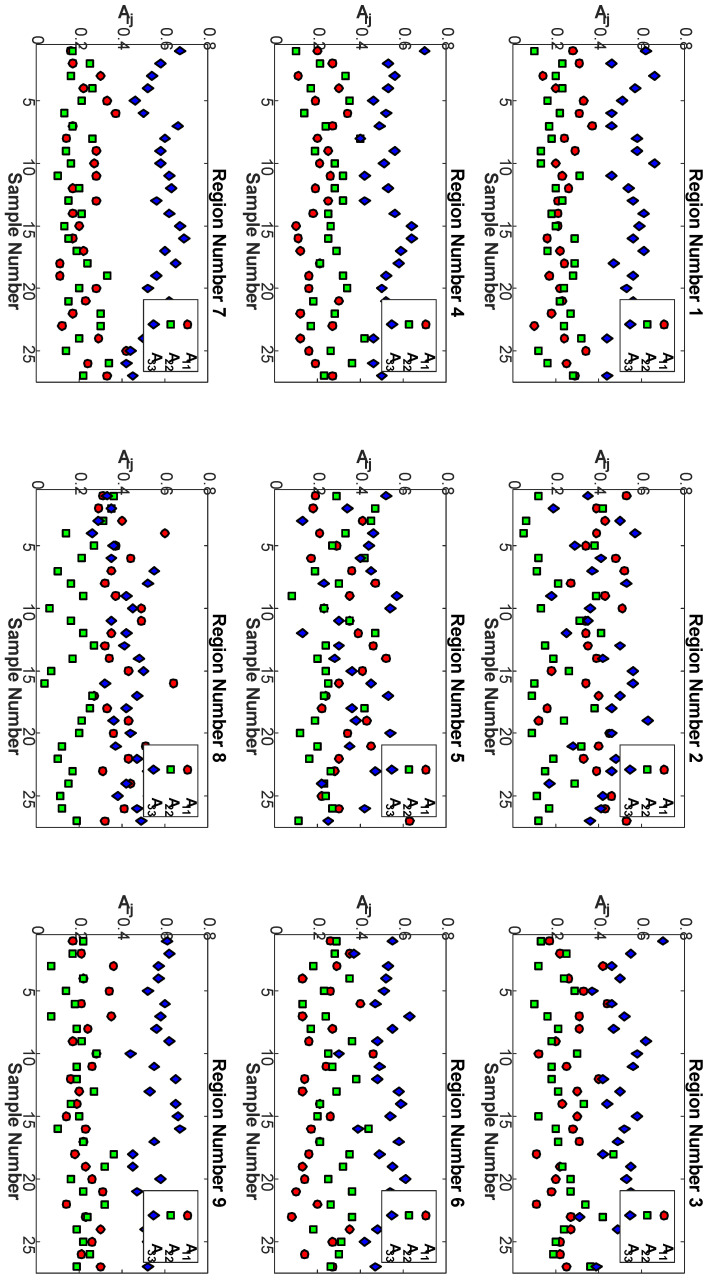
Plots of $A_{11}$, $A_{22}$, and $A_{33}$ for each sample by region.

The above process of orientation extraction from each of the nine regions for the remaining 27 samples is shown in Table 1 for the three different temperatures, screw speeds, and cross-sectional areas. The orientation results from all 27 samples for each of the nine regions are shown in Figure 10. In general, the trend that the alignment is highest along the print direction is shown by the increased value of $A_{33}$. The observation that the alignment is highest on the corners, Regions 1, 3, 7, and 9, is generally true, as is the observation that the $x_{3}$ alignment is lowest in the center of the deposited bead, Region 5. It is worth noting that there is an observed correlation between the fiber count and the fiber alignment along $x_{3}$ of a particular region.

### 4.1. Effect of Nozzle Temperature on Fiber Orientation

**Figure 11:**
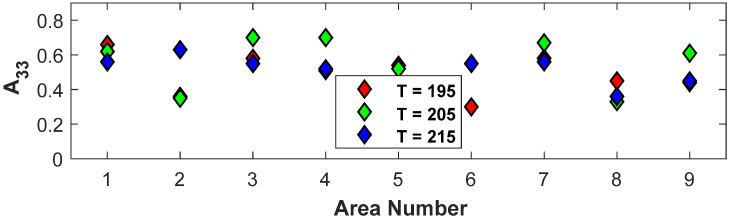
Change in orientation tensors for 1500 RPM and A=22 mm^2^ for varying temperature.

The sensitivity of the alignment to the processing parameter of temperature is investigated in the present section. This was accomplished by fixing the screw speed to 1500 RPM and the cross-sectional area to 22 mm^2^ (samples 1, 10, and 19 from Table 1). The variation in orientation tensors $A_{11}$, $A_{22}$_,_ and $A_{33}$ in each of the nine regions of Figure 2 for the changing nozzle temperature was computed, and $A_{33}$ is shown in Figure 11. Notice that the three temperature zones of 190/195/200 °C, 200/205/210 °C, and 210/215/220 °C have been noted as 195 °C, 205 °C, and 215 °C, respectively, in Figure 11. Overall, the temperature state of T=205 °C has the highest overall alignment, but this is by a nominal amount.

### 4.2. Effect of Screw Speed on Fiber Orientation

**Figure 12:**
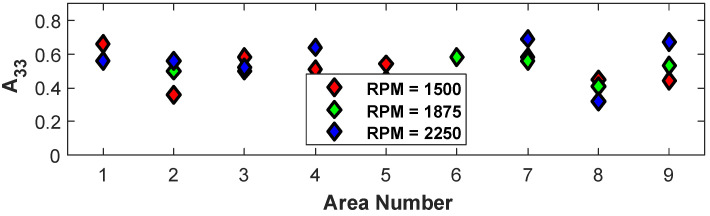
Change in orientation tensors for temperature = 190/195/200 °C and A=22 mm^2^ when varying the extrusion speed.

The sensitivity of the alignment to the processing parameter of screw speed is investigated in the present section. This was accomplished by fixing the temperature to 190/195/200 °C and the cross-sectional area to 22 mm^2^ (samples 10, 13, and 16 from Table 1). The variation in the orientation tensors $A_{11}$, $A_{22}$_,_ and $A_{33}$ in each of the nine regions depicted in Figure 2 by changing the screw speed to 1500 RPM, 1875 RPM, and 2250 RPM was computed, and the value for $A_{33}$ is shown in Figure 12. From the figure, it is not clear which of the three extrusion speeds results in the highest orientation state.

### 4.3. Effect of Cross-Sectional Area on Fiber Orientation

**Figure 13:**
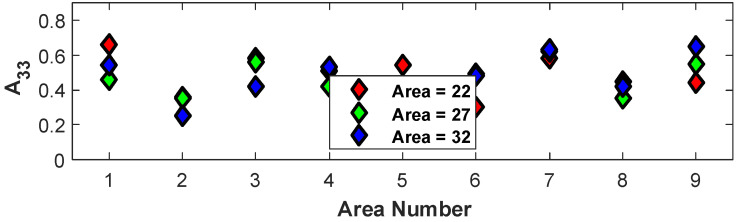
Change in orientation tensors at 9 regions with varying cross-sectional area with RPM = 1500 at nozzle temperature = 190/195/200 °C.

The sensitivity of the alignment to the processing parameter of the deposition area is investigated in the present section. This was accomplished by fixing the temperature to 190/195/200 °C and the extruder RPM to 1500 RPM (samples 10, 11, and 12 from Table 1). The variation in the orientation tensors $A_{11}$, $A_{22}$_,_ and $A_{33}$ in each of the nine regions depicted in Figure 2 by changing the deposition area from 22 mm^2^, 27 mm^2,^ and 32 mm^2^ was computed, and the value for $A_{33}$ is shown in Figure 13. From the figure, it is not clear which of the three cross-sectional areas yields the highest orientation state.

### 4.4. Correlation among All Three Process Parameters with the Orientation Tensors

**Figure 14:**
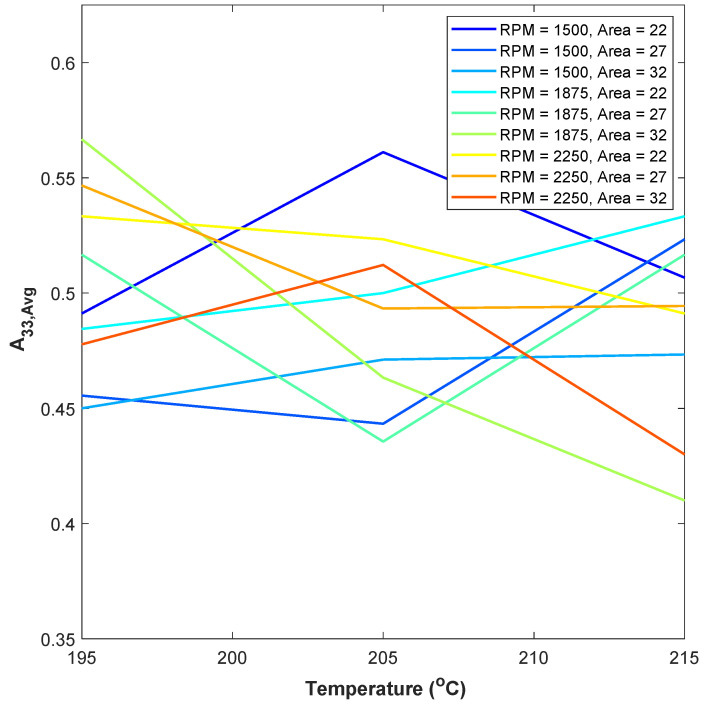
Change in *A*_33_ value for different print conditions.

The orientation state from all 27 samples from Table 1 is presented in Figure 14. The value presented is the orientation taken from a single sample by averaging the orientation from the nine regions within the sample. The error bars are not plotted in the figure as they provide confusion, but for each sample, the typical standard deviation is ~0.074.

From Figure 14, it can be seen that the two highest alignments of fibers in the $x_{3}$ direction occur for the print condition with the temperature of 200/205/210 °C, screw speed of 1500 RPM, and cross-sectional area of 22 mm^2^ and the print condition with the temperature of 190/195/200 °C, screw speed of 1875 RPM, and cross-sectional area of 32 mm^2^.

Figure 14 shows a trend of the fiber orientation being the greatest towards the print direction at lower nozzle temperatures generally at higher screw speeds. Again, for the lowest RPM and lowest cross-sectional area, and the highest RPM and highest cross-sectional area, the fiber orientation towards the print direction can be obtained at the nozzle temperature of 200/205/210 °C.

Studying and analyzing all 27 samples by changing three process parameters, it can be concluded that there is not a statistically significant correlation between the investigated process parameters and the orientation tensors. This is unfortunately not a strong conclusion as to the sensitivity of the fiber orientation to processing parameter variation.

### 4.5. Fiber Orientation Measurement for Different Samples with Identical Processing Conditions

**Figure 15:**
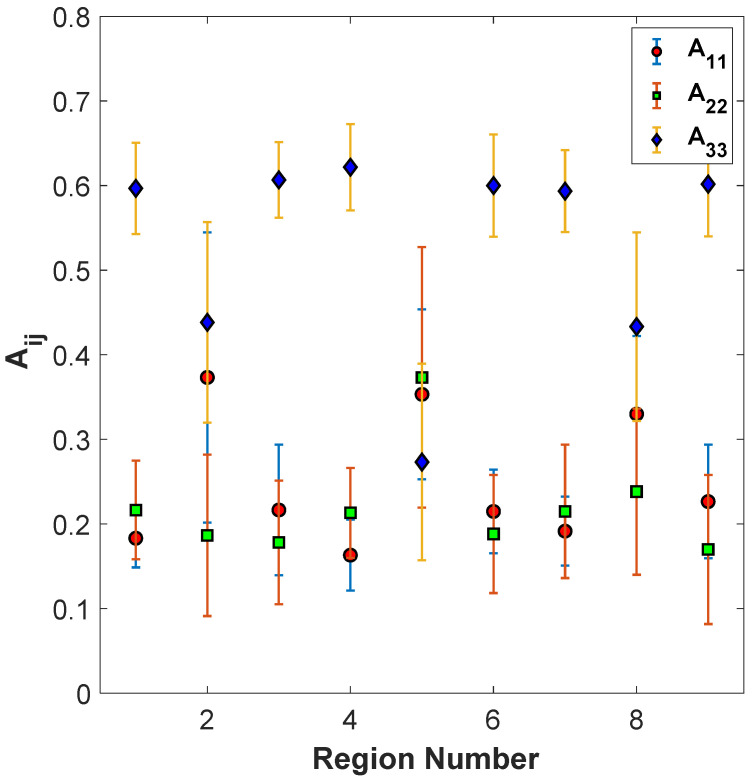
Plots of the average of $A_{11}$, $A_{22}$, and $A_{33}$ for 6 samples taken from deposited beads fabricated with the properties of Part #15.

This final study takes multiple cross-sections of the same deposited bead to identify any inhomogeneities within the bead itself. A single deposited bead is sectioned at six locations along the bead, and then the orientation tensors $A_{11}$, $A_{22}$, and $A_{33}$ are measured using the aforementioned approach for SEM image construction. In Figure 15, the average of the orientation tensors for the nine regions along the six samples is shown. These six samples are printed at a nozzle temperature of 190/195/200 °C, a screw speed of 1875 RPM, and a cross-sectional area of 32 mm^2^, the same parameters as sample 15 from Table 1. The mean orientation from each region is plotted in Figure 15, with the error bars defined as one standard deviation about the mean. There is a significant variation in the value of the tensors along the length of the printed part, in contradiction to the assumed state of uniformity along the deposited bead length. The $A_{33}$ component within each region varies across each of the six samples.

It can be observed from Figure 15 that the orientation tensor values follow the same trend as discussed before of having the highest values in the print direction on the corners (Regions 1, 3, 7, and 9) and on the edges (Regions 4 and 6), with the near-the-surface regions along the deposition center line (Regions 2 and 8) having a reduced alignment and the center of the deposited bead (Region 5) having the lowest alignment state. As shown by the error bars, taken from the respective standard deviations, the orientation tensor values in the same region for the samples taken along the same bead have considerable variation. Thus, there is an expected considerable amount of part-to-part variation. This is in alignment with the results from the preceding sections where there are few conclusions that can be made in regard to the processing parameter sensitivity. Thus, to properly correlate the sensitivity of processing parameters to the orientation state, additional sectioned samples would be required along the length of individual beads, and this was beyond the scope of the present study. Based on the results in Figure 15, it is clear there is significant variability along the extruded bead for the same process parameters, and before a quantification can be made to identify the optimal set of process parameters, the manufacturing process itself must be made more consistent.

## 5. Conclusions and Future Work

The present paper presented a method to quantify the spatial variation in the orientation state of carbon fibers within an additively manufactured deposited bead. For this study, 13% weight fraction carbon fiber-reinforced ABS pellets were printed using a custom large area additive manufacturing (LAAM) system over a range of processing parameters. The resulting extrudate was sectioned, polished, and subsequently analyzed with SEM for studying spatial variations of the local fiber orientation. The presented work introduced a novel approach using scanning electron microscopy to image sectioned samples to quantify the orientation state. Methods for proper cutting, polishing, cleaning, and plasma etching of the sample are presented in this work. The proper preparation of the samples, including etching, was shown to be helpful in providing a high contrast of the fiber–matrix interface.

The variation in fiber alignment was analyzed by changing three different process parameters: nozzle temperature, screw speed, and cross-sectional area of the printed beads. A total of 27 samples were printed, and each sample was subdivided into nine regions to study the spatial variation in fiber orientation. The results presented in this study indicate that fibers are most aligned at the corners of the extrudate, with the fibers in the center region of the extrudate being near a random orientation state. There are subtle variations in the sensitivity of the orientation to variations in the process parameters from scan to scan, but drawing conclusions is made difficult due to the finite sample sizes and the variability within the same sample caused by the limited available data. To quantity this latter issue, six samples from the same processing parameter set were fabricated, sectioned, imaged, and characterized. From this large dataset, it was seen that a better estimation of the mean values could be obtained with a standard deviation between samples of greater than 0.05 for the longitudinal component of the orientation tensor $A_{33}$. This value is significant as the range of the orientation tensor component of the average $A_{33}$ over the 27 permutations of process parameters is between 0.42 and 0.57. Thus, for example, the process parameter set of RPM=1500, A=22 mm^2^, and T=205 ℃ had the highest value of alignment with $A_{33}=0.57$, but 9 of the remaining 26 sets of investigated process parameters exhibited an alignment within one standard deviation of the highest average alignment measured.

**Figure 16:**
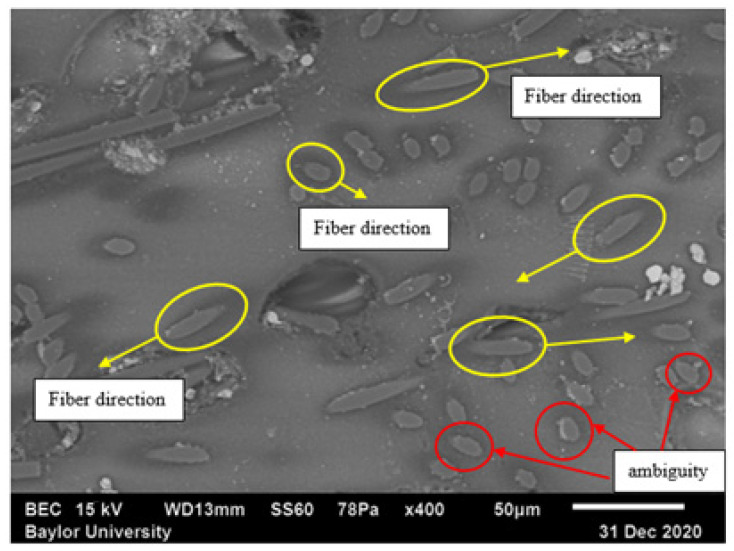
Tracing under the surface shadow of fiber to determine the orientation (Region 5 of sample 15). Fibers highlighted in yellow indicate representative fiber alignment that might be properly characterized using optical microscopy, whereas the fibers highlighted in red have a clear SEM shadow but would be ambiguous using optical microscopy and standard polishing techniques.

As part of the developed method, an interesting future study was revealed by the nature of the electron charge distribution build-up on the surface. This may provide a solution to the ambiguity problem inherent to the method of ellipses. For example, Region 5 of sample 15 is selected to present this potential solution and is shown in Figure 16. Observe that SEM generates a three-dimensional-like image of the surface. In the SEM image, an under-surface shadow of each fiber can be traced as noted by the non-uniform darkened regions around the fiber; thus, the actual orientation of each fiber in the matrix from Figure 7 can be determined. In the figure, several fibers are highlighted, and the resulting dark-gray tail can be observed. It is hypothesized that this dark-gray region is caused by the semi-conducting nature of the carbon fibers, thus allowing the surface change to dissipate in a mechanism different from that of the polymer surface, an insulating material. This is especially obvious in fibers with a higher elliptical nature for which the fiber under the surface remains closer to the surface for a larger distance.

In the future, more samples need to be studied to establish the correlations of fiber orientation to the investigated processing parameters. Regardless, the orientation state for the investigated printed composite was quantified, and a moderate degree of alignment was observed. Additional work will be required in manufacturing to optimize the alignment state as well as to promote consistency within the deposited beads themselves. It is noted that there were a considerable number of voids in all samples, and it is well established that void formation in fiber-filled systems tends to reduce the structural performance while randomizing the orientation state. Thus, it is suggested that an investigation of the sensitivity of the final void content to processing parameters may yield more promise in achieving the goal of increasing the alignment state.

## Figures and Tables

**Table 1:** Characterization of sample number according to the process parameters.

		Screw Speed 1500 RPM	Screw Speed 1875 RPM	Screw Speed 2250 RPM
Temperature Zones 190/195/200 (°C)	Area 22 mm^2^	10	13	16
	Area 27 mm^2^	11	14	17
	Area 32 mm^2^	12	15	18
Temperature Zones 200/205/210 (°C)	Area 22 mm^2^	1	4	7
	Area 27 mm^2^	2	5	8
	Area 32 mm^2^	3	6	9
Temperature Zones 210/215/220 (°C)	Area 22 mm^2^	19	22	25
	Area 22 mm^2^	10	13	16
	Area 27 mm^2^	11	14	17

**Table 2:** Orientation tensor for different regions of sample 15.

Region	1	2	3	4	5	6	7	8	9
Fiber Count	83	64	77	86	53	75	80	54	91
A11	0.21	0.18	0.3	0.1	0.41	0.26	0.2	0.43	0.14
A22	0.2	0.26	0.12	0.26	0.24	0.2	0.13	0.07	0.2
A33	0.59	0.56	0.58	0.64	0.36	0.54	0.67	0.5	0.66

## Data Availability

SEM images can be provided upon request to the corresponding author.
